# Clinical and serological follow-up of patients with WDEIA

**DOI:** 10.1186/s13601-019-0265-8

**Published:** 2019-05-16

**Authors:** Morten J. Christensen, Esben Eller, Charlotte G. Mortz, Knut Brockow, Carsten Bindslev-Jensen

**Affiliations:** 1 Department of Dermatology and Allergy Centre, Odense Research Center for Anaphylaxis (ORCA), Odense University Hospital, 5000 Odense C, Denmark; 2 Department of Dermatology and Allergy Biederstein, Technische Universität München, Munich, Germany

**Keywords:** Anaphylaxis, Wheat dependent exercise induced anaphylaxis, Gluten, oral food challenge, Exercise challenge, Specific IgE, Specific IgG, Avoidance

## Abstract

The clinical threshold in wheat-dependent, exercise-induced anaphylaxis seems to be lowered in patients on wheat free diet, whereas the opposite is seen in patients on regular wheat intake. Therefore, a recommendation of wheat consumption, if considered safe to the patient based on case-history and challenge results, could be advised.

**Electronic supplementary material:**

The online version of this article (10.1186/s13601-019-0265-8) contains supplementary material, which is available to authorized users.

To the Editor,

The current recommendations in managing food allergy focuses on avoidance of the offending allergen(s) and use of rescue medication in the event of an allergic reaction [1]. Induction of oral tolerance or desensitization is a potential preventive approach and small amounts of the allergenic food may induce faster tolerance, although the underlying immunological mechanism is incompletely understood [2–4].

Wheat-dependent, exercise-induced anaphylaxis (WDEIA) is a severe variant of food allergy caused by physical exercise or incompletely other co-factors in combination with ingestion of a food containing wheat [5, 6]. Based on case-history and challenge results patients are recommended either total wheat avoidance or to avoid exercise 4 h before and after wheat ingestion [7], since the underlying pathophysiological mechanisms of WDEIA are still incompletely understood [8].

The aims of this study were to evaluate the threshold and the immunoglobulin levels in two groups of patients with challenge confirmed WDEIA prescribed either total wheat avoidance or avoidance only in connection to physical exercise. We investigated 12 patients with WDEIA divided in two groups; total wheat avoidance (n = 5) and wheat consumption unless 4 h before or after exercise (n = 7), and conducted two titrated gluten challenges in combination with treadmill exercise 4 weeks apart during this time interval [5]. Skin prick test (SPT) with wheat and gluten together with measurement of specific-IgE (sIgE), sIgG and sIgG_4_ in serum to wheat and omega-5 gliadin using ImmunoCap (ThermoFisher Scientific, Uppsala, Sweden) were performed at the 1st and 2nd challenge and 4 weeks hereafter, respectively.

We used the statistical software program STATA^®^ version 15.0 (StataCorp LLC, College Station, Texas, USA) and applied the Wilcoxon signed ranks test for the intra-group analysis and Mann–Whitney tests for between-group analysis in the absolute change in threshold. Statistical significance was defined as a p value of less than 0.05. The study was approved by The Regional Committees on Health Research Ethics for Southern Denmark (Project ID: 20140012).

Among the 12 patients included, 8 were previously diagnosed by oral food challenge (OFC) with gluten ± exercise and for the remaining 4 patients they had their diagnostic challenge performed as part of the 1st challenge. At time of diagnosis different individual recommendations concerning gluten avoidance were applied corresponding to case-history and challenge results. The median time from the diagnostic challenge to the 1st challenge was 364 days (range 0–944 days). No significant change in the gluten threshold eliciting a reaction between the initial challenge and the 1st challenge was found (p = 0.44). There was no difference in clinical reactivity at rest between the two groups; in the total avoidance group 4/5 reacted with a median threshold of 25 g [8–80 g] compared to 5/7 in the consumption group with a median threshold of 58 g [24–80 g]. No reaction at rest was elicited at a maximum amount of 80 g of gluten in the avoidance group (n = 1) and consumption group (n = 2).

In the avoidance group (1 female and 4 male; mean age 51.8 years) the time interval between 1st and 2nd challenge was 35 ± 11 days and in 60% (3/5, 2 unchanged) a decrease of the threshold dose was observed with a median decrease of 3.2 g [0–12 g] (p = 0.09). In the consumption group (3 female and 4 male; mean age 50.7 years) the time interval between the challenges was 28 days ± 3 and the threshold dose increased in 71% (5/7, 2 unchanged) with a median increase of 9.6 g [0–32 g] (p = 0.05) (see Fig. 1). A significant difference was observed (p = 0.018) in the absolute change in thresholds between the avoidance group and the consumption group from the 1st to the 2nd challenge (see Fig. 1).

**Fig. 1:**
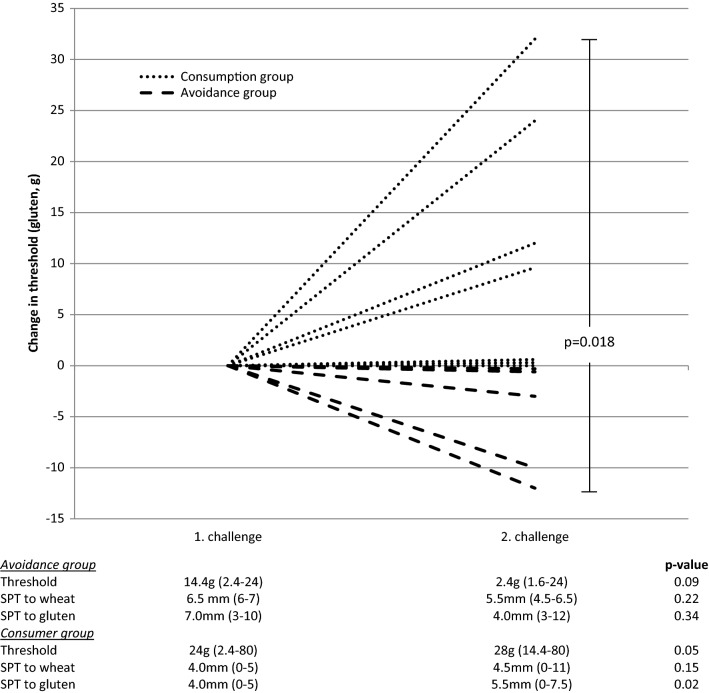
Change in threshold from 1st to 2nd challenge in groups of wheat avoidance and consumption. In the wheat avoidance group (n = 2) and the wheat consumption group (n = 3) had an unchanged clinical threshold between the 1st and 2nd challenge. Values of threshold and SPT are given as medians

Levels of sIgE, sIgG and sIgG_4_ to wheat and omega-5 gliadin in serum were measured at 1st and 2nd challenge as well as 4 weeks hereafter. There were no significant differences between groups or changes over time, neither for immunoglobulin levels, nor for the ratio of wheat or omega-5 gliadin sIgG_4_:IgE in any of the groups (see Additional file 1: Table S1). Wheal size with wheat and gluten did not change over the period between 1st and 2nd challenge (see Fig. 1).

Wheat is a source of food widely used in the daily household and this is to the author’s knowledge the first study comparing the clinical effects and immunological responses to groups with wheat avoidance and consumption in WDEIA. Although only patients with severe reactions and low thresholds were actively prescribed total avoidance, some patients autonomously chose total wheat avoidance for convenience. There is therefore no active randomization applied to the patients. There were no significant difference in rates of hospitalization, serious adverse events or exercise frequency/intensity between the avoidance group and the consumption group during study period.

The main finding is that patients with doctor guided total avoidance developed a decrease in threshold and those on regular wheat intake outside 4 h before or after exercise had an increase in threshold levels, which was not reflected by skin test and immunoglobulin parameters. Therefore, regular intake of wheat not related to exercise may be superior as compared to total avoidance for building up higher threshold levels and may be recommended. This could be similar to oral immunotherapy in children with wheat allergy. Results have shown successful development of tolerance accompanied by increased levels of sIgG_4_ and decreased levels IgE to wheat, 6 months after an up-dosing regimen [9]. Surprisingly, an expected increase in levels of sIgG_4_ or sIgG elicited by challenge was not seen in any of the two groups, nor was the level of sIgG in serum higher in the consumption group compared to the avoidance group, in contrast to regular successful immunotherapy, where an increase in sIgG and IgG_4_ is seen [10]. This may be related to the lack of high allergen doses given during immunotherapy and to the short duration of the study of only 2 months.

A weakness to this study is a relatively small study size of 12 patients with a lack of a blinded randomized regimen and that in both groups before the 1st challenge, either wheat avoidance or wheat consumption was allowed. Furthermore additional dose-titration-steps of gluten (maximum 4 steps) could have been applied in other to determine and demonstrate a wider change in threshold in the respective groups.

In conclusion, the clinical threshold in WDEIA seems to be lowered in patients on wheat-free diet, whereas the opposite is seen in patients on regular wheat intake. Therefore, a recommendation of wheat consumption, if considered safe to the patient based on case-history and challenge results, could be chosen. Further investigations and larger investigations are however needed to support these findings.

## Additional file

**Additional file 1: Table S1.** Levels of sIgE, sIgG and sIgG_4_ to wheat and omega-5 gliadin in groups of wheat avoidance and consumption in WDEIA. * 1 patient and 2 patients in the wheat avoidance group and the wheat consumption group, respectively, did not have a blood sample drawn 4 weeks after the 2nd challenge.

## Data Availability

Authors can confirm that all relevant data are included in the article and/or its supplementary information files.

## Abbreviations

sIgE: specific immunoglobulin E
OFC: oral food challenge
SPT: skin prick test
WDEIA: wheat-dependent exercise-induced anaphylaxis
