# Supplementary material for: Clinical and serological follow-up of patients with WDEIA
Source: Clin Transl Allergy. 2019 May 16;9:26. doi: 10.1186/s13601-019-0265-8 (PMC6524276; doi:10.1186/s13601-019-0265-8)
Supplement: Supplementary file 1 — Additional file 1: Table S1. Levels of sIgE, sIgG and sIgG4 to wheat and omega-5 gliadin in groups of wheat avoidance and consumption in WDEIA. * 1 patient and 2 patients in the wheat avoidance group and the wheat consumption group, respectively, did not have a blood sample drawn 4 weeks after the 2nd challenge. [file 13601_2019_265_MOESM1_ESM.docx]

|  |  |  |  |  |
| --- | --- | --- | --- | --- |
|  | **1. challenge** | **2. challenge** | **4 weeks after** | **p-value** |
| *Avoidance group** |  |  |  |  |
| sIgE wheat | 0.74 (0.29-2.8) | 0.6 (0.51-1.88) | 1.35 (0.26-3.5) | ns. |
| sIgE omega-5 gliadin | 10.1 (3.7-36.6) | 9.5 (4.5-36.3) | 10.5 (3.9-44.9) | ns. |
| sIgG wheat | 7.3 (5.5-13.5) | 8.2 (4.2-13.1) | 10.7 (4.1-12.8) | ns. |
| sIgG omega-5 gliadin | 2.6 (2.2-4.5) | 2.5 (2.1-4.4) | 3.1 (2.1-4.2) | ns. |
| sIgG_4_ wheat | 0.19 (0.14-2.6) | 0.23 (0.15-2.7) | 0.7 (0.13-3.2) | ns. |
| sIgG_4_ omega-5 gliadin | 0.07 (0.01-0.11) | 0.05 (0.02-0.07) | 0.06 (0.04-0.07) | ns. |
| sIgG_4_/sIgE wheat | 0.28 (0.05-4.0) | 0.28 (0.08-4.4) | 2.0 (0.09-3.9) | ns. |
| sIgG_4_/sIgE omega-5 gliadin | 0.006 (0-0.022) | 0.005 (0.001-0.016) | 0.006 (0.001-0.019) | ns. |
| *Consumer group*** |  |  |  |  |
| sIgE wheat | 0.46 (0.08-3.7) | 0.46 (0.07-3.8) | 2.08 (0.06-6.1) | ns. |
| sIgE omega-5 gliadin | 6.3 (0.9-15.1) | 5.9 (1.0-14.2) | 11.8 (1.4-20.7) | ns. |
| sIgG wheat | 7.8 (3.4-12.7) | 8.2 (3.8-12.3) | 7.7 (3.8-14.4) | ns. |
| sIgG omega-5 gliadin | 2.0 (1.0-3.0) | 1.7 (1.3-2.2) | 2.2 (1.3-2.3) | ns. |
| sIgG_4_ wheat | 0.6 (0.04-3.0) | 0.7 (0.03-3.1) | 0.6 (0.06-3.5) | ns. |
| sIgG_4_ omega-5 gliadin | 0.04 (0.01-0.06) | 0.03 (0.02-0.2) | 0.03 (0.02-0.2) | ns. |
| sIgG_4_/sIgE wheat | 0.86 (0.17-11.3) | 1.16(0.19-14.1) | 0.58 (0.14-3.16) | ns. |
| sIgG_4_/sIgE omega-5 gliadin | 0.004(0.003-0.047) | 0.013(0.002-0.018) | 0.013 (0.002-0.017) | ns. |
|  |  |  |  |  |
